# Supplementary figures and images for: Accurate detection of shared genetic architecture from GWAS summary statistics in the small-sample context
Source: PLoS Genet. 2023 Aug 16;19(8):e1010852. doi: 10.1371/journal.pgen.1010852 (PMC10461826; doi:10.1371/journal.pgen.1010852)

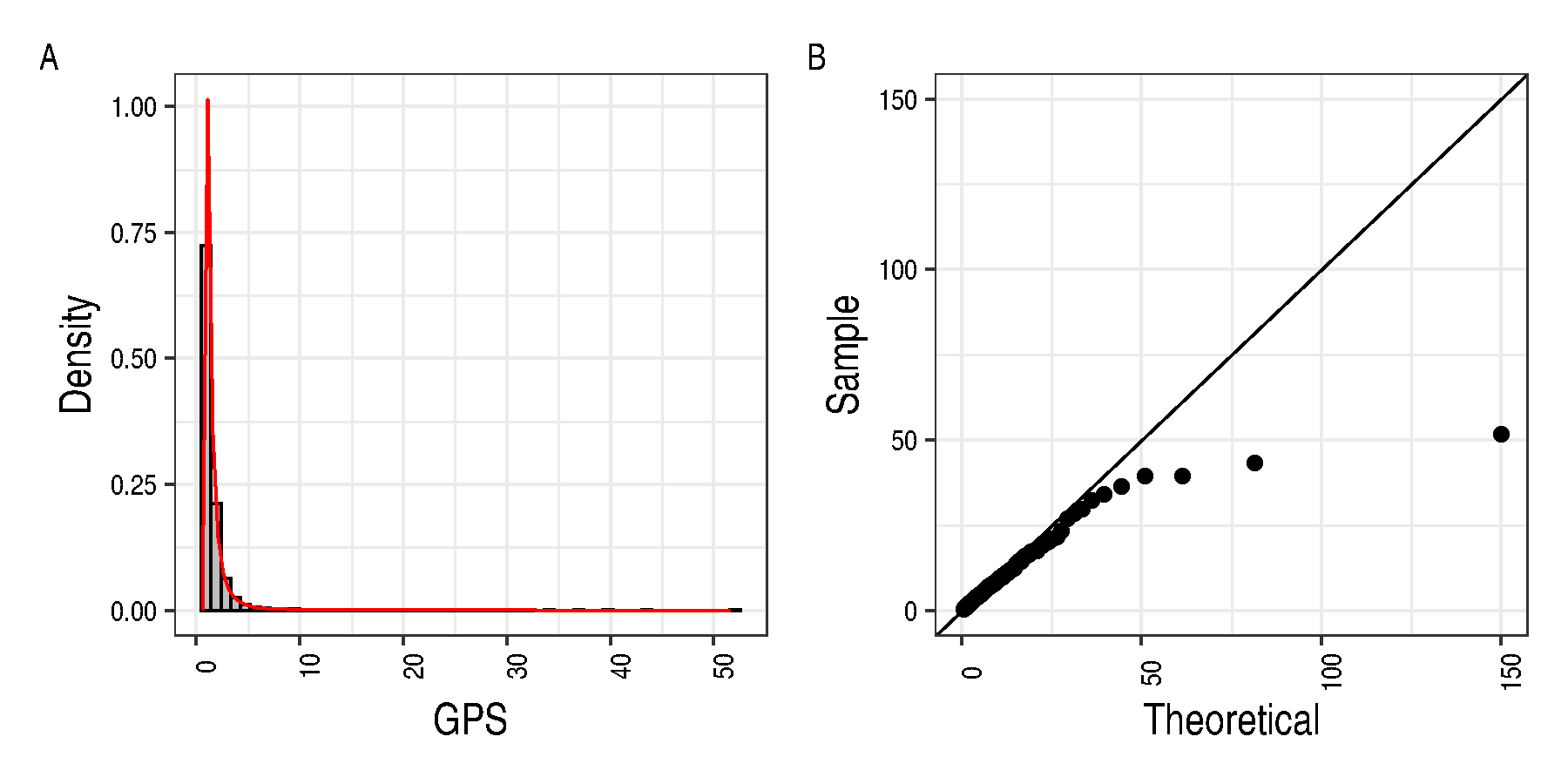

Supplement: S1 Fig — (A) a histogram of GPS statistics with fitted GEVD density superimposed as the red line and (B) a quantile-quantile plot of empirical (‘Sample’) and fitted GEVD (‘Theoretical’) quantiles. Null realisations of the GPS statistic were produced by permutation of the order of SNPs in GWAS of asthma and emphysema/chronic bronchitis from the UK Biobank collection. (TIF) [file pgen.1010852.s001.tif]

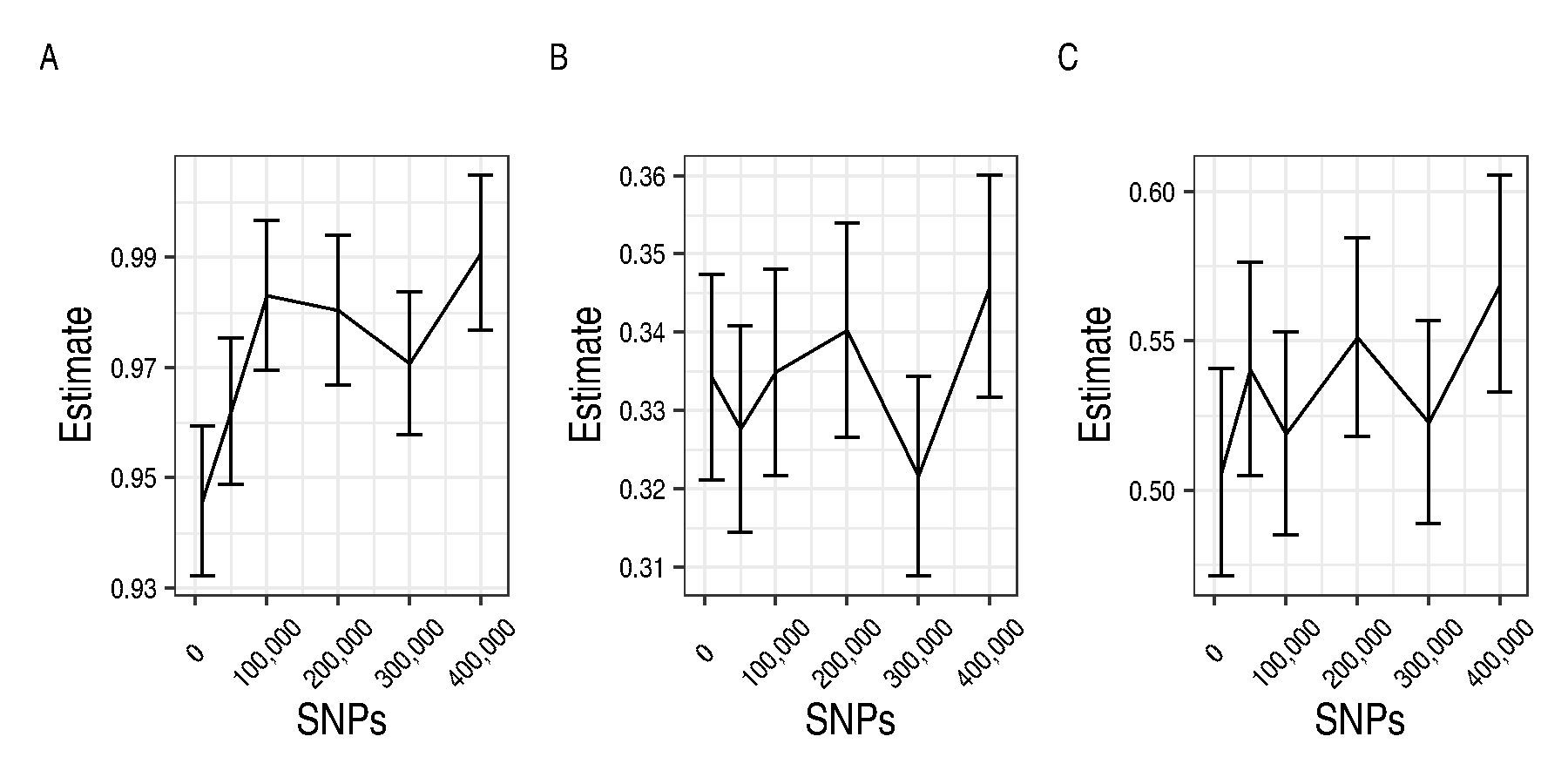

Supplement: S2 Fig — (A) The location parameter. (B) The scale parameter. (C) The shape parameter. The error bars depict 95% confidence intervals for each estimate. The estimates were obtained by permuting the order of SNPs in a pair of GWAS summary statistics data sets and downsampling these same data sets to obtain the specified number of SNPs. The estimates shown were obtained using GWAS of asthma and emphysema/chronic bronchitis in the UKBB data set. (TIF) [file pgen.1010852.s002.tif]

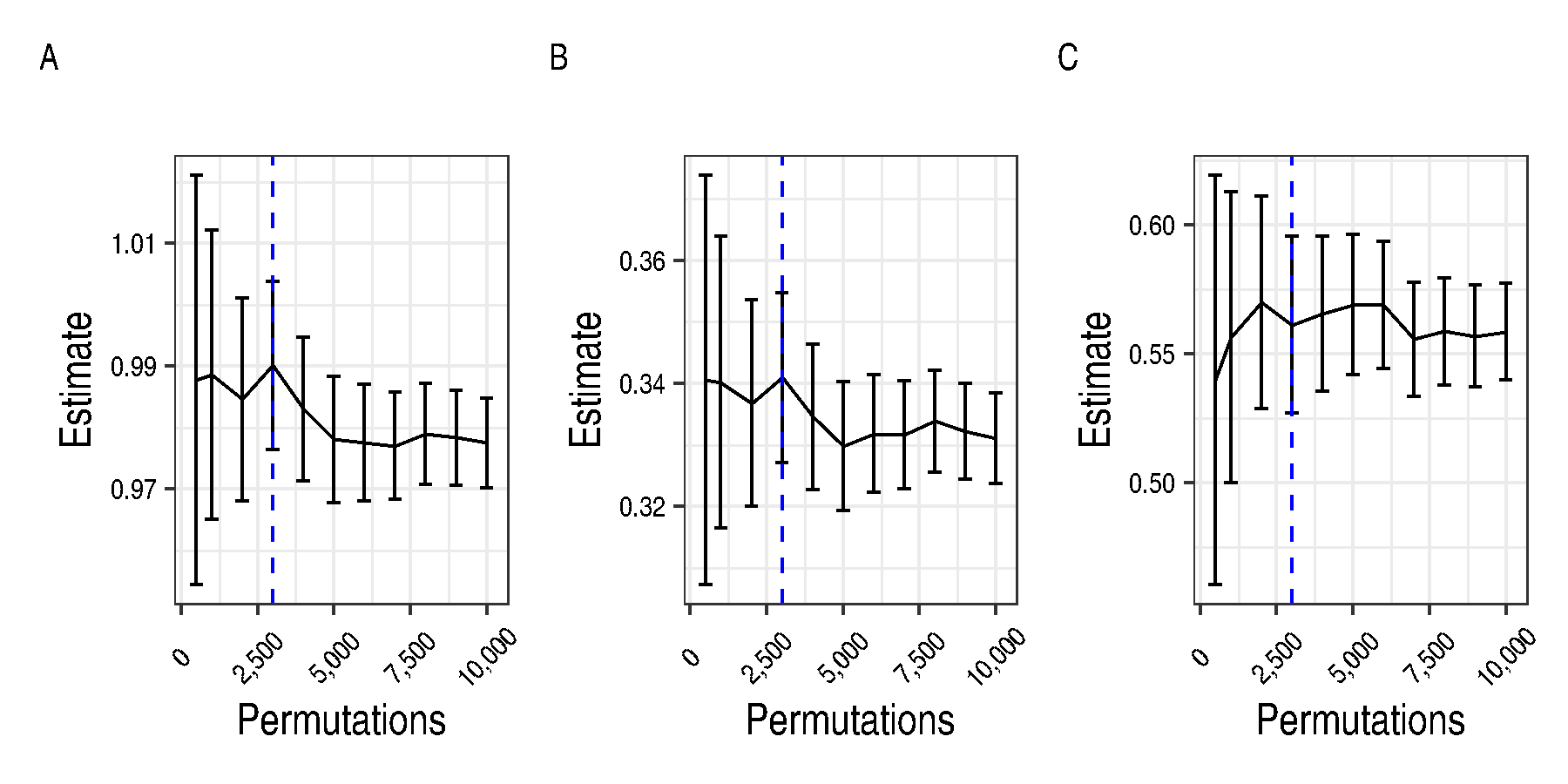

Supplement: S3 Fig — (A) The location parameter. (B) The scale parameter. (C) The shape parameter. Error bars depict 95% confidence intervals for each estimate. The dashed blue line indicates 3,000 permutations. The estimates were obtained by permuting the order of SNPs in a pair of GWAS summary statistic data sets. The estimates shown were obtained using GWAS of asthma and emphysema/chronic bronchitis. (TIF) [file pgen.1010852.s003.tif]

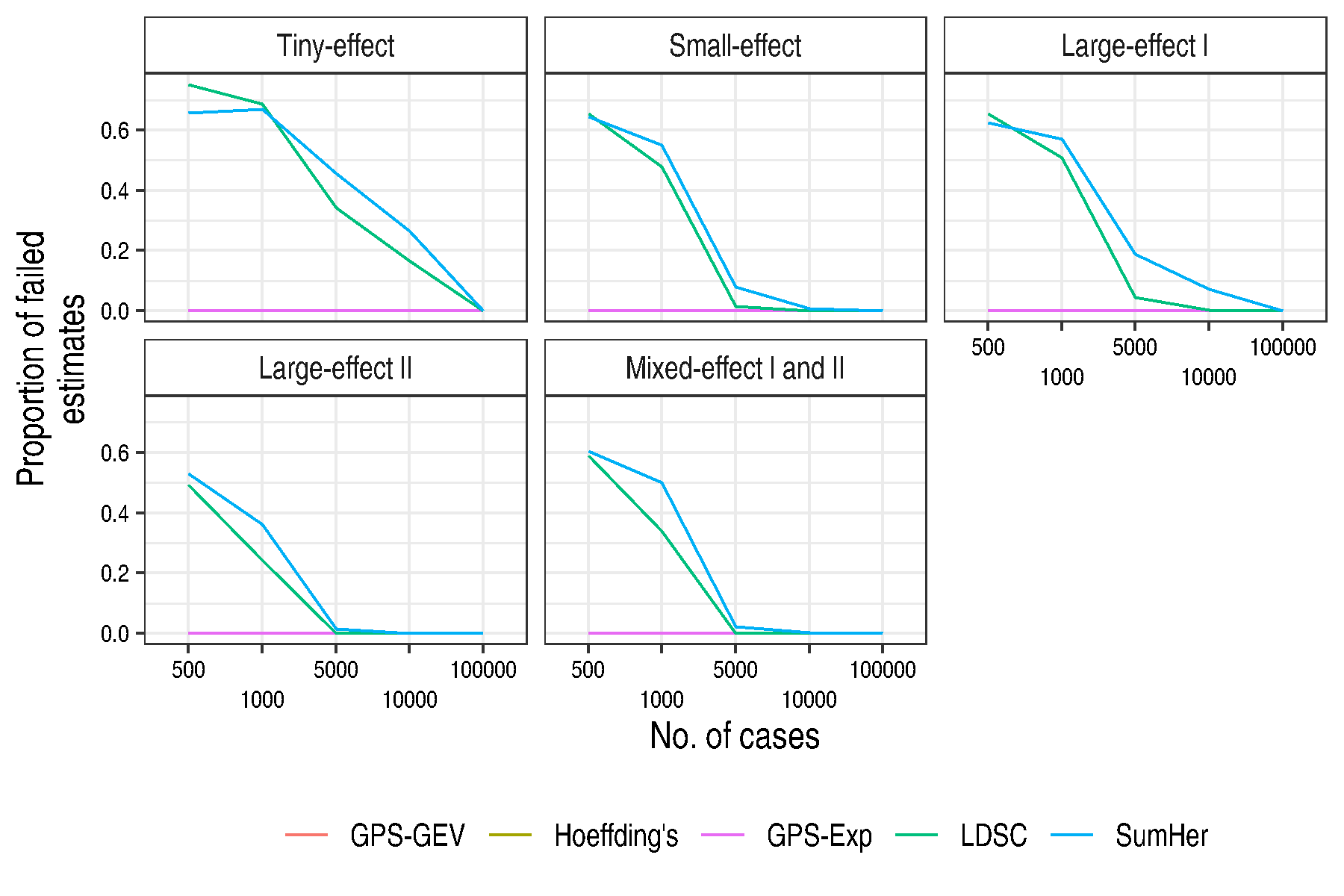

Supplement: S4 Fig — Failures were recorded only for LDSC and SumHer. (TIF) [file pgen.1010852.s004.tif]

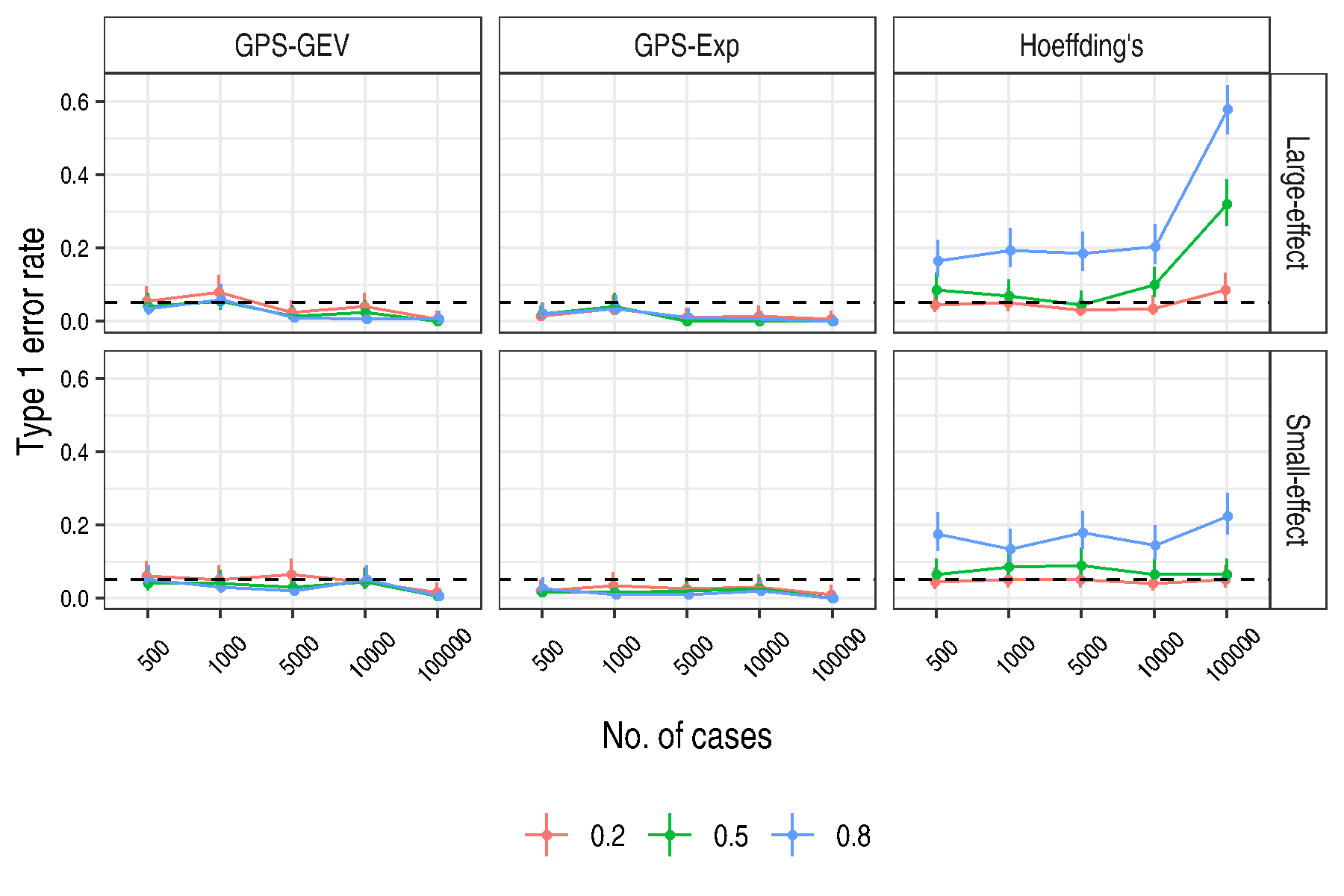

Supplement: S5 Fig — The type 1 error rate for each method was estimated as the proportion of replicates for which p ≤ 0.05 when the genetic correlation was set to zero. This proportion was measured among 200 replicates. 95% confidence intervals were calculated as Wilson score intervals. The dotted line depicts the size of the test, 0.05. (TIF) [file pgen.1010852.s005.tif]

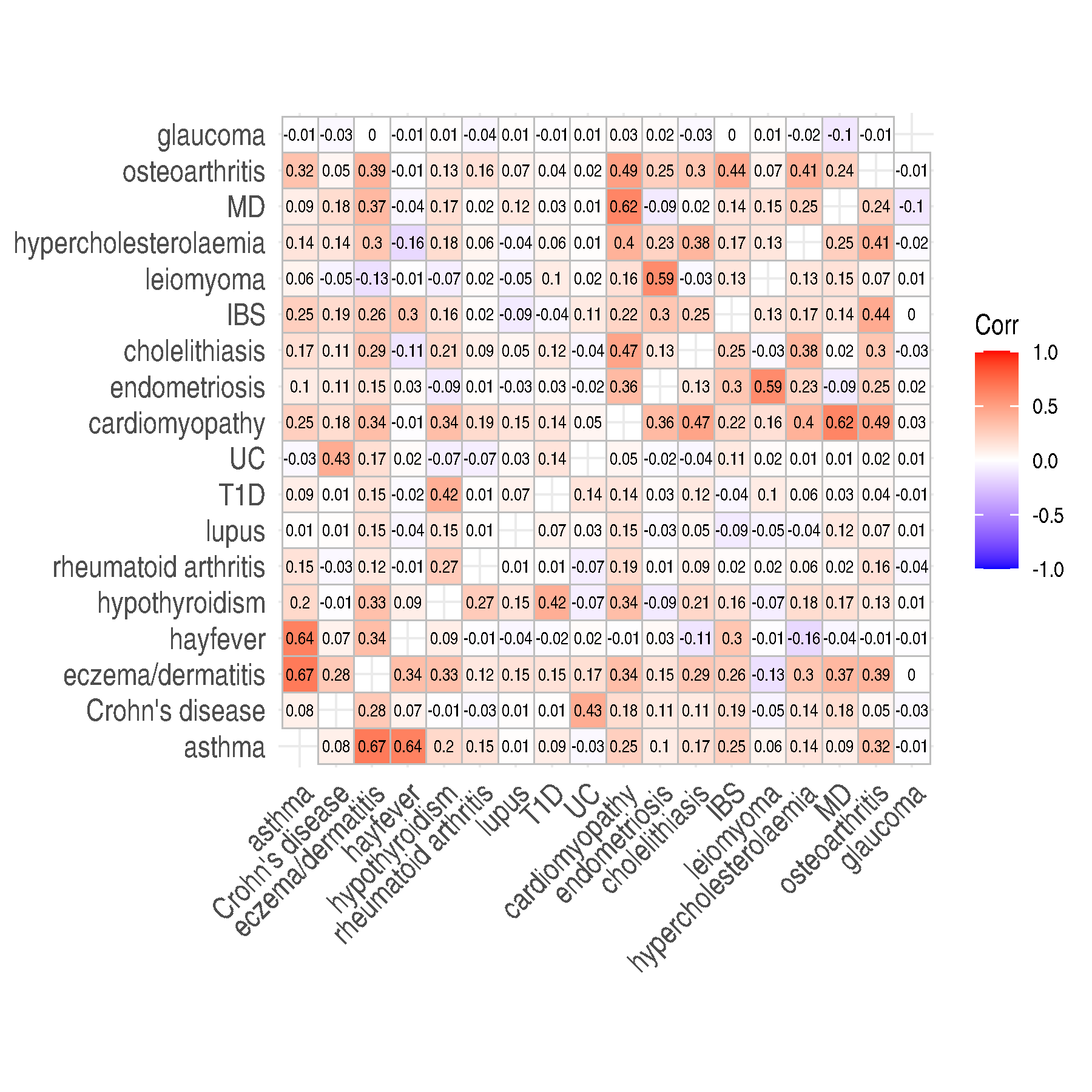

Supplement: S6 Fig — ‘Corr’ is estimated genetic correlation. (TIF) [file pgen.1010852.s006.tif]

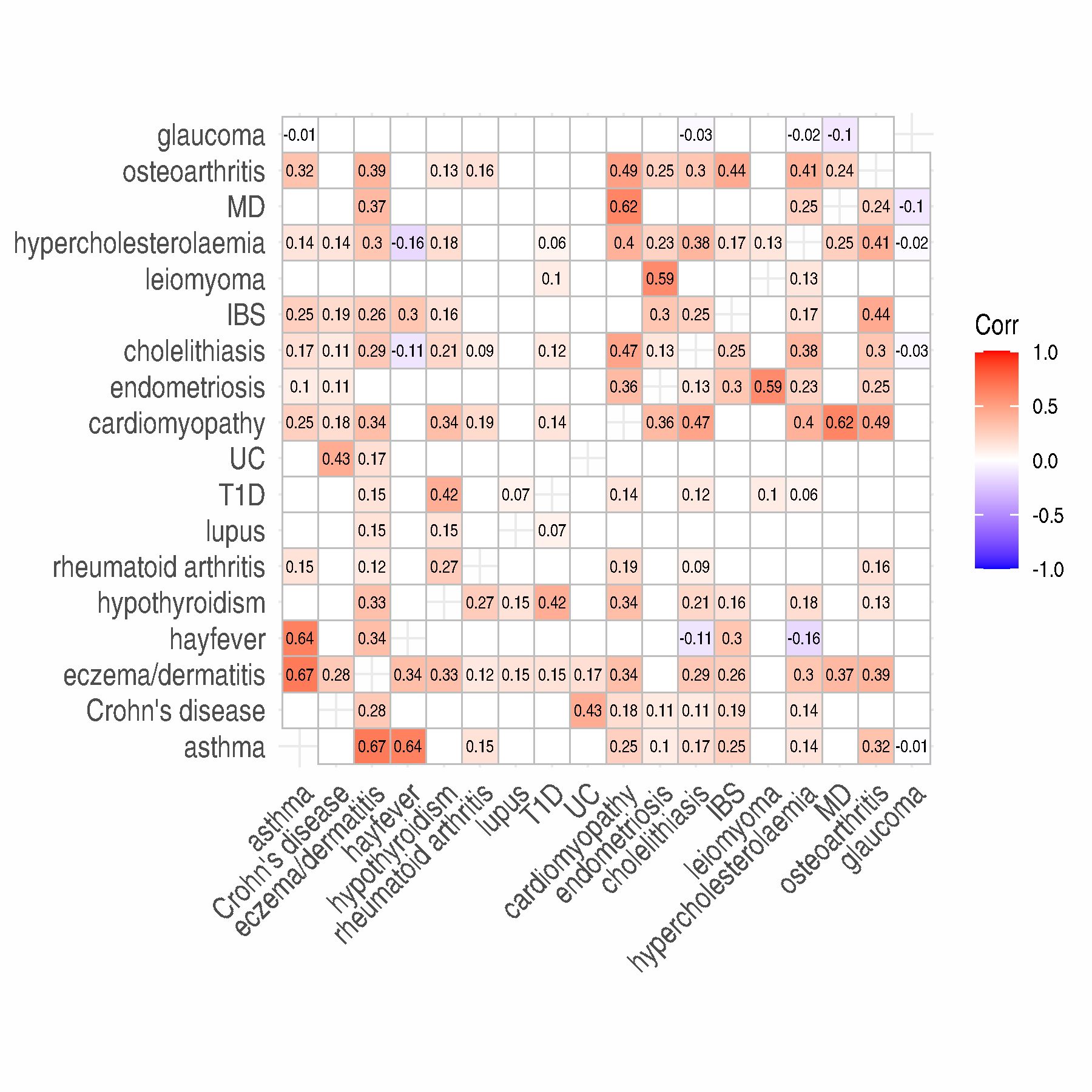

Supplement: S7 Fig — A nominally significant estimate was taken as one for which p ≤ 0.05 from a chi-squared test of non-zero genetic correlation. ‘Corr’ is estimated genetic correlation. (TIF) [file pgen.1010852.s007.tif]

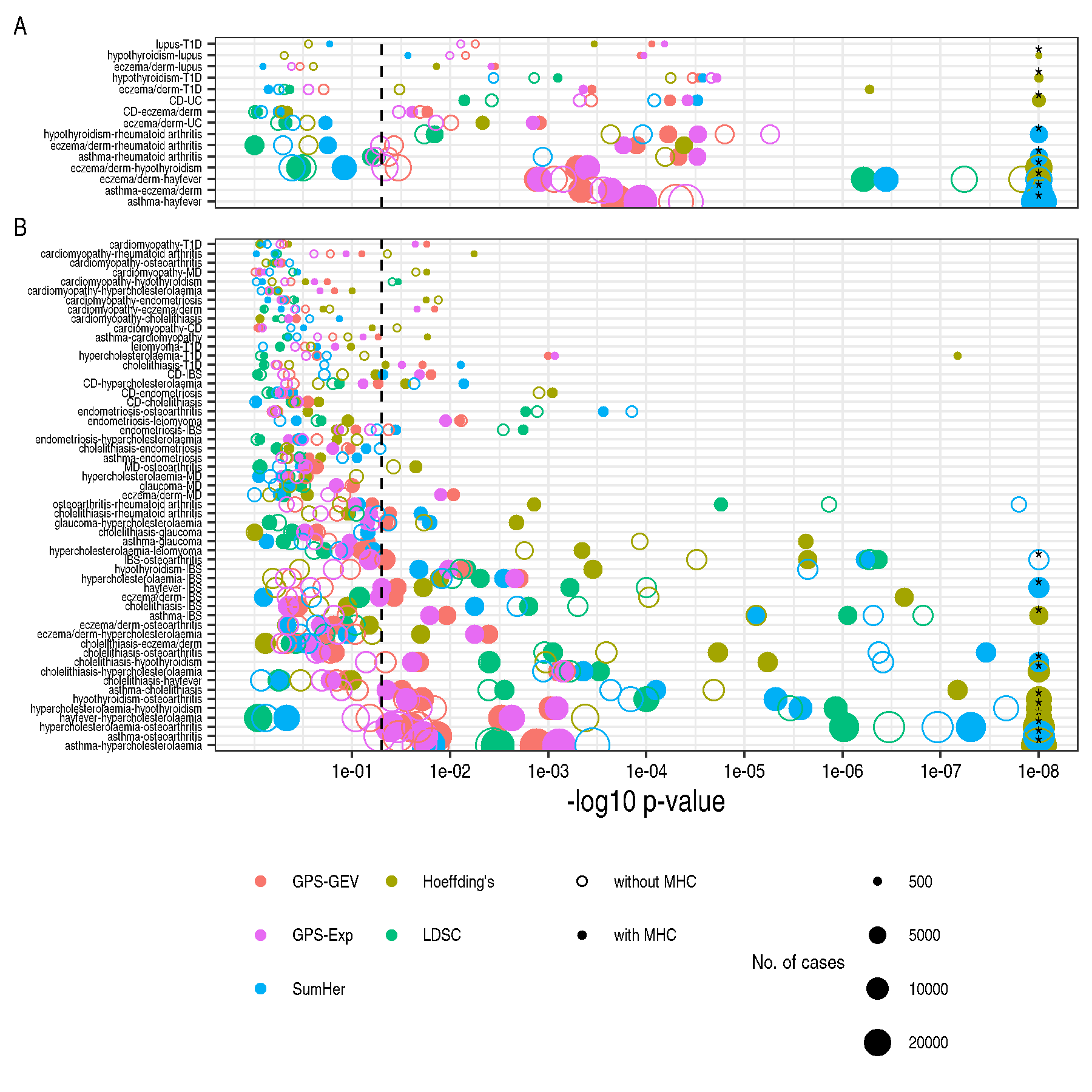

Supplement: S8 Fig — The ‘No. of cases’ gives in terms of point size the smaller number of disease cases in each pair of case-control GWAS. The dashed line lies at 0.05. Filled points indicate the use of the entire data set, hollow points indicate the use of a data set with the MHC removed. Where p-values were smaller than 1e-8, they have been aliased to 1e-8 and indicated with an asterisk. For very small case numbers, it was not possible to obtain genetic correlation estimates with LDSC and SumHer for some data sets. (TIF) [file pgen.1010852.s008.tif]

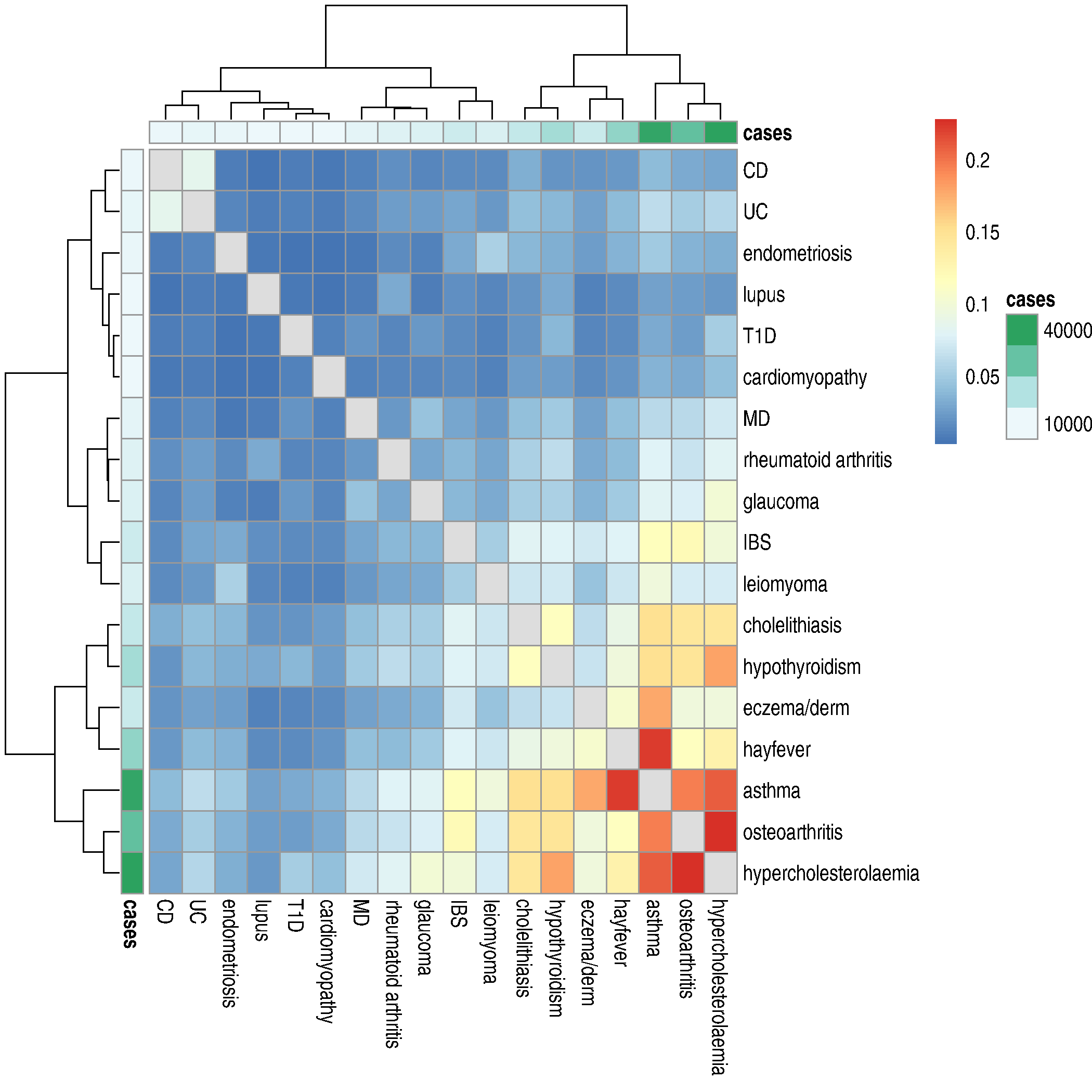

Supplement: S9 Fig — The shade of the heatmap gives the value of ρ for each pair of traits. The sample size of each GWAS is depicted in terms of its number of cases on the supplementary colour scale. (TIF) [file pgen.1010852.s009.tif]

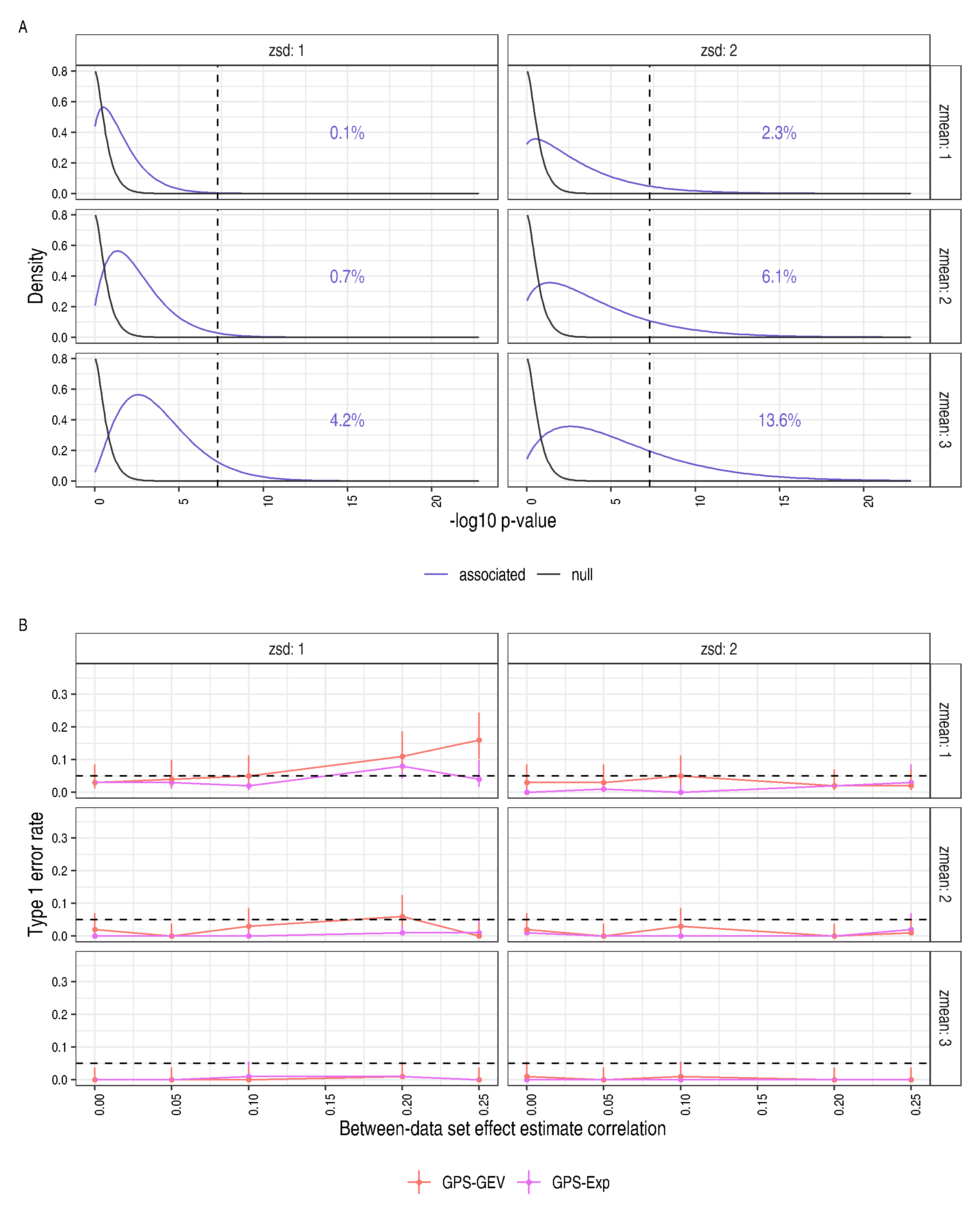

Supplement: S10 Fig — (A) GWAS p-values were simulated from a transformed mixture of normal distributions with null and non-null components. The distribution of null and non-null p-values is depicted in black and blue, respectively. ‘zmean’ and ‘zsd’ give the mean and standard deviation of the normal distribution from which non-null Z-score addends were drawn for each data set. The percentage of SNPs expected to exceed the genome-wide significance threshold of 5 × 10^−8^ for each simulation is stated; this point is depicted by the vertical dashed line. (B) The type 1 error rate for each simulation as ρ increases from 0 to 0.25. Type 1 error was estimated as the proportion of replicates for which p ≤ 0.05. 95% confidence intervals were calculated as Wilson score intervals. The dotted line depicts the size of the test, 0.05. (TIF) [file pgen.1010852.s010.tif]
