# Supplementary material for: Accurate detection of shared genetic architecture from GWAS summary statistics in the small-sample context
Source: PLoS Genet. 2023 Aug 16;19(8):e1010852. doi: 10.1371/journal.pgen.1010852 (PMC10461826; doi:10.1371/journal.pgen.1010852)
Supplement: S1 Text — (PDF) [file pgen.1010852.s011.pdf]

Accurate detection of shared genetic architecture from GWAS summary statistics in the small-sample context
S1 Text: Supplementary tables

Thomas W. Willis Chris Wallace

Table A. Summary statistics for timing benchmarks for candidate bivariate ecdf algorithms used in the GPS permutation procedure. All values are given in seconds (s).

ecdf algorithm	Minimum (s)	1st quartile (s)	Median (s)	Mean (s)	3rd quartile (s)	Maximum (s)
naive	4317.7	4570.3	4759.2	4710.8	4793.6	4956.6
Langrené and Warin	8.8	10.0	10.5	10.4	10.9	11.7
Perisic and Posse	3.8	4.9	5.0	5.0	5.2	5.6

Table B. The number of cases and controls, and case-control ratio in the five sample configurations used in our simulation studies.

No. of cases	No. of controls	Case-control ratio
500	10,000	0.05
1,000	10,000	0.1
5,000	10,000	0.5
10,000	10,000	1
100,000	100,000	1

Table C. The number of SNPs included in each analysis. GPS-Exp, GPS-GEV, and Hoeffding's test were applied to LD-pruned sets which varied with respect to the value of the r^2 parameter used in pruning and which is given in the header. For LDSC we report the number of SNPs used in regression after merging the real or simulated data set with the panel of precomputed LD scores (Text S2). For SumHer we report the number of SNPs after merging the real or simulated data set with taggings generated from Phase 3 1kGP data; this number is reported in SumHer log files as the number of predictors for which summary statistics were available (Text S2). We did not analyse the single-chromosome simulated data with LDSC or SumHer nor did we perform whole-genome analyses with $r^2 = 0.5$ or 0.8.

Data set	LDSC	SumHer	$r^2 = 0.2$	$r^2 = 0.5$	$r^2 = 0.8$
UKBB with MHC	1,178,572	9,487,152	591,015	1,524,514	2,722,772
UKBB without MHC	1,171,710	9,352,179	586,552	1,511,656	2,696,810
simGWAS whole-genome simulations	1,118,361	8,015,936	525,150	-	-
simGWAS chromosome 1 simulations	-	-	42,537	97,256	165,910

Table D. Details of the two single-chromosome simulation regimes.

Name	Odds ratio(s)	No. of causal variants	No. of shared causal variants
Large-effect (chromosome 1)	1.2	20	5, 10, 15, 20
Small-effect (chromosome 1)	1.05	60	15, 30, 45, 60

Table E. Details of the exemplary GWAS data sets. 'R5' denotes 'Data Freeze 5', a particular iteration of publicly released FinnGen summary statistics.

Trait	Category	No. of cases	No. of controls	Collection	Citation
lupus	immune	5,201	9,066	-	[2]
type 1 diabetes	immune	18,942	501,368	-	[4]
Crohn's disease	immune	12,194	28,072	-	[5]
ulcerative colitis	immune	12,366	33,609	-	[5]
rheumatoid arthritis	immune	19,234	61,565	-	[6]
eczema/dermatitis	immune	20,052	198,740	FinnGen (R5)	[1]
hypothyroidism	immune	26,064	192,728	FinnGen (R5)	[1]
hayfever	immune	27,419	416,137	Pan-UKBB	[3]
asthma	immune	56,065	389,965	Pan-UKBB	[3]
cardiomyopathy	non-immune	11,400	175,752	FinnGen (R5)	[1]
endometriosis	non-immune	8,288	68,969	FinnGen (R5)	[1]
macular degeneration	non-immune	3,794	419,170	Pan-UKBB	[3]
glaucoma	non-immune	8,591	210,201	FinnGen (R5)	[1]
leiomyoma	non-immune	18,060	105,519	FinnGen (R5)	[1]
irritable bowel syndrome	non-immune	11,159	425,748	Pan-UKBB	[3]
cholelithiasis	non-immune	19,350	417,176	Pan-UKBB	[3]
osteoarthritis	non-immune	39,442	404,702	Pan-UKBB	[3]
hypercholesterolaemia	non-immune	47,737	394,138	Pan-UKBB	[3]

Table F. Median genetic correlation estimates by group and sample size as computed from the exemplary data sets. 'Mixed' pairs were those with one immune and one non-immune disease or two non-immune diseases. $\hat{r}_g$ denotes estimated genetic correlation. 'No. of cases' gives the smaller number of disease cases in each pair of case-control GWAS. We categorised pairs by their smaller case number in the UKBB collection, not the exemplary collection.

Group	No. of cases	No. of pairs	Median $\hat{r}_g$
Immune/immune	$\leq$ 2,000	21	0.07
Immune/immune	$>$ 2,000	15	0.15
Immune/immune	Any	36	0.09
Mixed	$\leq$ 2,000	54	0.11
Mixed	$>$ 2,000	63	0.07
Mixed	Any	117	0.09

References

1. Kurki MI, Karjalainen J, Palta P, Sipilä TP, Kristiansson K, Donner K, et al.. FinnGen: Unique Genetic Insights from Combining Isolated Population and National Health Register Data; 2022.

2. Bentham J, Morris DL, Cunninghame Graham DS, Pinder CL, Tombleson P, Behrens TW, et al. Genetic Association Analyses Implicate Aberrant Regulation of Innate and Adaptive Immunity Genes in the Pathogenesis of Systemic Lupus Erythematosus. Nature Genetics. 2015;47(12):1457–1464. doi:10.1038/ng.3434.

3. Pan-UKB team. Pan-UK Biobank; 2020.

4. Chiou J, Geusz RJ, Okino ML, Han JY, Miller M, Melton R, et al. Interpreting Type 1 Diabetes Risk with Genetics and Single-Cell Epigenomics. Nature. 2021;594(7863):398–402. doi:10.1038/s41586-021-03552-w.

5. de Lange KM, Moutsianas L, Lee JC, Lamb CA, Luo Y, Kennedy NA, et al. Genome-Wide Association Study Implicates Immune Activation of Multiple Integrin Genes in Inflammatory Bowel Disease. Nature Genetics. 2017;49(2):256–261. doi:10.1038/ng.3760.

6. Okada Y, Wu D, Trynka G, Raj T, Terao C, Ikari K, et al. Genetics of Rheumatoid Arthritis Contributes to Biology and Drug Discovery. Nature. 2014;506(7488):376–381. doi:10.1038/nature12873.
