# Supplementary material for: Accurate detection of shared genetic architecture from GWAS summary statistics in the small-sample context
Source: PLoS Genet. 2023 Aug 16;19(8):e1010852. doi: 10.1371/journal.pgen.1010852 (PMC10461826; doi:10.1371/journal.pgen.1010852)
Supplement: Attachment — Submitted filename: response.pdf [file pgen.1010852.s015.pdf]

Reviewer 1

Willis and Wallace propose an improved version of the GPS test. This tests for pleiotrophy between pairs of traits (i.e., whether they have a common genetic architecture). GPS was first proposed (by different authors) in 2015. In this paper, Willis and Wallace explain a fundamental "flaw" ijn the original version of the test, and also provide a solution. They then compare GPS to alternative methods for detecting shared genetic architecture (e.g., LDSC and Sumher) on both simulated and real data, finding examples where GPS is more powerful.

Overall, I found this a easy paper to read (I thought it was well-written). I considered the modification mathematically sound, and the applications of the method generally extensive. This is not a ground breaking paper, in that the GPS method is not hugely used, and it has limitations (particularly, the sensitivity to individual SNPs, and the lack of an interpretable parameter), all of which are acknowledged by the authors. Nonetheless, the authors do a good job finding situations where it might be useful, and provide extra insight (in particular, for rare traits where there are very few samples). Further, the GPS can always be used (unlike LDSC or Sumher that sometimes fail).

Major comments

I have no major comments. To me the method seemed mathematically sound, and as I say, the authors fairly described its utility and shortcomings.

Minor Comments

1 - Please can you make numbers of SNPs more prominent in the paper. I could not see it specified in the main text. Further, it as also hard to find in the methods (e.g., you described in detail the pruning of 1000G data, but did not mention how many snps remained). For example, perhaps you could add under results a mention that you "usually use LD-pruned data, so n is typically 40k-80k", or whatever the correct numbers are. This would also be useful as computation time presumably is about linear in number of SNPs, so it would make your claim of 1 hour on 8 CPUs more informative.

We now give the number of SNPs where apposite in the text and have added a table to Text S1 to summarise the number of SNPs in the simulated and UK Biobank (UKBB) data sets, as well as the number used as input to each method (Table S3).

Since our first submission, we have improved our implementation of the GPS test statistic by making use of Perisic and Posse's order statistic-based algorithm, which we implemented in C++, for the computation of bivariate ecdfs. The GPS statistic can now be computed twice as quickly as when we first submitted our manuscript. The time complexity is O(n log n) in n, the number of SNPs.

We added a paragraph to Verification and comparison discussing the computation time required by each GPS test (including this new implementation) with reference to the number of SNPs in the input:

> "To determine how best to balance the need for accurate estimates against the cost of computation, we benchmarked three bivariate ecdf algorithms, the computation of the bivariate ecdf being by far the most costly step in calculating the GPS statistic. These were the naive algorithm making use of two nested loops, Langrené and Warin's recently published divide-and-conquer algorithm, and an order

statistic tree-based algorithm from Perisic and Posse. We found the last of these to be the fastest, offering a near-1000-fold advantage in speed over the naive algorithm and a 2-fold advantage over Langrené and Warin's algorithm in the bivariate case (Table S1). The formulation of the permutation procedure is 'embarrassingly parallel' and we exploited this with a multithreaded implementation. Computing 3,000 GPS permutations for a 525,150-variant data set across 12 cores in parallel with Perisic and Posse's algorithm took approximately 18 minutes (or 3 hours and 17 minutes in CPU time). We chose 3,000 permutations as a compromise between estimate accuracy and running time. By way of comparison, the calculation of the GPS-Exp test p-value is computationally trivial, taking less than one second of CPU time."

2 - When describing the simulations, you mention cases numbers, but please can you also mention case control ratio (e.g. do you always have equal cases and controls, or is total sample size fixed). My understanding is that "small-sample" primarily refers to number of cases, rather than small overall sample size. Similarly, you had this sentence "The GPS test identified genetic similarity among IMD pairs across a range of sample sizes" - whereas I felt you were mainly considering different case numbers.

Our principal interest was in the effect of the number of cases upon power; a small number of cases is the typical difficulty with which we contend in our own study of rare diseases and our motivation for using the GPS test in the first place. We have endeavoured to make this clearer in the Materials and methods section of Text S2 as follows:

"We also varied the number of cases and controls across five sample configurations (Table S2). As cases are typically in far shorter supply than controls in case-control GWAS, we sought to emulate this by choosing a greater number of controls, 10,000, than cases for the first three sample configurations where the number of cases was less than 10,000. For the next two configurations with 10,000 and 100,000 cases, respectively, we chose a matching number of controls."

We have added a supplementary table to Text S1 as a point of reference for the different sample configurations and have included a column specifying case-control ratio (Table S2):

Table S2. The number of cases and controls, and case-control ratio in the five sample configurations used in our simulation studies.

No. of cases	No. of controls	Case-control ratio
500	10,000	0.05
1,000	10,000	0.1
5,000	10,000	0.5
10,000	10,000	1
100,000	100,000	1

3 - I was confused why the total sample size varied for UKBB traits (especially if you got them from Neale Lab, whom I thought tried to use as many samples as possible). Please can you add a quick comment (sorry if I missed it).

As per our new passage in the Materials and methods section of Text S2:

"The Neale lab included a total of 361,194 biobank samples in their GWAS analyses but case/control status with respect to each (binary) phenotype was not defined for all samples (http://www.nealelab.is/uk-biobank/faq), such that the sum of cases and controls varies across our chosen phenotypes (Table 2)."

There is some redundancy among the phenotypes present in the Neale lab collection, so we have added a column specifying UK Biobank phenotype code to Table 2 to make clear precisely which phenotype we have used for each trait.

4 - It might be useful to report phenotypic similarity (or average across the four classes, immune/immume, immune/non-immune, more than 2k, less than 2k).

We have computed out-of-sample, 'exemplary' genetic correlation estimates for the diseases we considered by assembling an additional collection of large GWAS, one for each disease chosen from the UK Biobank. Median genetic correlation as estimated from these data sets by group and case number is now presented in Table S6:

Group	No. of cases	No. of pairs	Median $\hat{r}_g$
Immune/immune	$\leq$ 2,000	21	0.07
Immune/immune	> 2,000	15	0.15
Immune/immune	Any	36	0.09
Mixed	$\leq$ 2,000	54	0.11
Mixed	> 2,000	63	0.07
Mixed	Any	117	0.09

These exemplary data sets included FinnGen and Pan-UK Biobank studies, and thus some cases and controls were shared between them.

We restructured our real data set analyses with the use of the exemplary genetic correlation estimates. We used them to identify 15 immune/immune pairs for which the estimates were significantly different from 0 ($p < 0.05$) to serve as surrogate true positives with which to evaluate the power of each test on real data. In addition, we identified 62 mixed pairs (i.e. pairs with at least one non-immune disease) for which the estimates were *not* significantly different from 0 to serve as surrogate true negatives with which to evaluate each test's control of type 1 error. We summarise these results in Table 3 (the entries show the percentage of nominally significant pairs for each method):

Group	MHC	No. of cases	Median $\hat{r}_g$	No. of pairs	GPS-GEV	GPS-Exp	Hoeffding's	LDSC	SumHer
immune	✓	$\leq 2,000$	0.15	7	100	100	86	29	43
immune	✓	$> 2,000$	0.30	8	100	100	100	50	62
immune	✗	$\leq 2,000$	0.15	7	71	71	43	29	29
immune	✗	$> 2,000$	0.30	8	100	75	75	50	63
mixed	✓	$\leq 2,000$	0.02	30	30	30	37	13	17
mixed	✓	$> 2,000$	0.01	32	34	28	50	6	6
mixed	✗	$\leq 2,000$	0.02	30	3	3	27	10	3
mixed	✗	$> 2,000$	0.01	32	9	0	38	3	6

5 - Figure 3 - I assume red line is present in bottom right panels (but cant be seen) - is there some way to make it visible, or perhaps put red line on top (because we care about that method most)

We have added 'jitter' to the x-axis values for this plot to more clearly separate the points and have brought the GPS-GEV line to the front (Figure 3).

6 - You compare your new version of GPS with other tools. Is it worth also including the original version of GPS (although you have shown it is flawed, it seems it is "conservatively" flawed? - ie tends to give false negatives, rather than false positives, so a comparison would show the improvement in power of your modifications)

We now include the original GPS test (referred to as the ‘GPS-Exp test’ on the basis of its Exp(1) null distribution) as a comparator for our updated GPS test (the ‘GPS-GEV’ test). This was a worthwhile exercise and has shown that the GPS-GEV test offers an advantage in power on real and simulated data sets.

7 - There are more details in LDSC and SumHer than most realise. For example, default LDSC estimates genetic correlations across about 8M sequenced snps with MAF>0.005, whereas its default estimates of SNP heritability are across about 4M sequenced SNPs with MAF>0.05 (which in my view is a contradiction, it considers rare SNPs problematic when it comes to SNP heritability, but fine when it comes to GC (or inflation)).

Similarly, for SumHer, estimates depend on choice of heritability model (which you explained), but also on choices of “regression SNPs, heritability SNPs and reference SNPs”. Note that I believe you used fewer reference snps for Sumher than LDSC, which might explain its higher power (because fewer reference SNPs leads to slightly smaller SDs)
In theory, you could pick these settings to best match your simulated data - ie, work out the assumed heritability model, and the most appropriate regression SNPs, heritability SNPs and reference SNPs. Then your results from LDSC / SumHer would be the best available.

I do not recommend doing this, because in practice, you do not know the truth.

So in summary, I dont think it is necessary / appropriate to go into these technical details (even though they can be important). But instead, I would just acknowledge something along the lines that you consider both LDSC and SumHer because they make contrasting assumptions (in particular, regarding genetic architecture), and thus it is reassuring to see they both perform similarly

We express our gratitude for this insightful explanation. We in fact used more SNPs with SumHer than with LDSC due to our use of a panel of precomputed LD scores with MAF > 0.05 for the latter as directed on LDSC’s GitHub page. We have added this information in Table S3.

We now describe the distinction between LDSC and SumHer briefly in Verification and comparison:

> “To assess the ability of the two GPS tests to detect genetic similarity, we compared them to tests of non-zero genetic correlation using LDSC and SumHer. The heritability models we use for genetic correlation estimation with these tools make contrasting assumptions with respect to genetic architecture. The LDSC model considers only common SNPs with minor allele frequency (MAF) > 0.05 and assumes that per-SNP heritability is uniform across these SNPs; this uniform model, also termed the ‘GCTA’ model, is to be distinguished from the higher-dimensional LDSC models used in heritability estimation outside of the genetic correlation estimation context. By contrast, per-SNP heritability in SumHer’s ‘LDAK-Thin’ model varies as a function of MAF and the level of linkage disequilibrium (LD).”

Some very minor comments (no responses required)

I thought your description and use of my software Sumher was accurate, many thanks. I also liked the introduction (the mention of hairless dogs, blue-eyed cats was a fun change from the generic introductions that most of us are guilty of writing). On the other hand, I prefer “without” to “sans”.

We agree and have duly made the change from ‘sans’ to the less pretentious ‘without’.

Reviewer 2

The authors propose a method to access if genetic correlation between two phenotypes is significantly different from zero, by using a modified version of a non-parametric test introduced by Li et al. (2015). The authors argue that an extreme value distribution should be assumed under the null, which is the modification of Li et al. (2015) who used a transformation of the standard exponential distribution. The authors used simulated data as well as real data (GWAS of 18 phenotypes from the UK Biobank) to show that the proposed method outperforms existing genetic correlation methods when using less polygenic traits with a small sample size.

Main comments

This paper is interesting, but there are a number of questions and comments that should be positively addressed.

Eq. (1) is mostly identical with that introduced by Li et al. (2015) (reference #37 in this paper). Eq. (2) is the distribution used in Li et al. (2015). What is the equation used in this study? I am not very clear if the proposed method is substantially improved from the original method or not.

The statistic in Equation (1) is indeed identical to that introduced by Li et al, i.e. the GPS statistic we compute is the same as that computed for the original GPS test. While we did not include the p-values realised from the use of Equation (2) in our original submission, we now do, and term this test “the GPS-Exp test”. We have added a third equation, Equation (3), to explicitly specify the generalised extreme value (GEV) distribution we propose as a better null distribution for the statistic and we refer to the resulting p-values as coming from the GPS-GEV test. As well as the practical evaluation of the GPS across different scenarios of (co-)heritability, a more accurate estimate of the statistic’s null distribution is the main theoretical contribution of this work.

The authors argue that they establish that the null distribution of the test statistic is better modelled by an extreme value distribution than a transformation of the standard exponential distribution. However, they did not really compare the performances for the original and proposed methods. What are type I error rate and power when using a transformation of the standard exponential distribution, and how they are compared with the results shown in Figures 2 and 3 (possibly Table 3)?

We have added the original GPS test to our comparisons; we label it the GPS-Exp test to distinguish it from the GPS-GEV test we propose. The GPS-Exp test is now included in our studies of type 1 error (Figure 2) and power (Figure 3) in simulated data sets and in our study of the tests applied to real UK Biobank data sets (Table 3 and Figure S8). Both statistics control the type 1 error rate, while we found a modest advantage for the GPS-GEV over the GPS-Exp in power.

I am not sure why the type error rates are deviated from the expectation when sample size increases for GPS (Fig. 2). For other methods, it is also unexpected that some data points are significantly deviated from the expectation.

The observation that type 1 error rate declines with increasing sample size is an insightful one and motivated us to study the phenomenon. In brief, we found that the null distribution

estimated by our permutation procedure did not match the null distribution of the GPS statistic observed empirically from directly simulated data at very large sample sizes, leading to misestimation of the GEV distribution's parameters (in particular the scale parameter) and an overly conservative GPS-GEV test. We found that the GPS-Exp test exhibited the same behaviour, albeit due to a poorer fit of the standard exponential distribution to null data at higher sample sizes. This is a shortcoming of both GPS tests which we were not able to overcome.

This conservative behaviour was observed at very large sample sizes rarely seen in existing case-control GWAS, even among those studies originating from biobanks and large consortia like the Psychiatric Genomics Consortium. As discussed in the manuscript, we envision use of the GPS test in the small-sample environment; were 100,000 cases available, parametric approaches such as those of LDSC and SumHer would likely be preferred to the GPS test due to the opportunity to estimate biologically meaningful parameters for such data. Nonetheless, we appreciate this comment for its prompting us to better characterise the GPS test.

We report our findings in Text S3 and have added a reference to this to the section in which we report our study of type 1 error rates in Verification and comparison.

In the legend of Fig. 2, it says, "This proportion was measured only among those replicates producing a valid test statistic." What does mean by a valid test statistic? How many replicates (%) are invalid and why? It is not clear if this caused the biased type I error (deflation). The authors should make sure the biased type I error rate is not because of this artificial process (excluding some replicates due to some numerical issue that may be correlated with type I error rate).

For simulated samples with smaller numbers of cases such as 500 and 1,000, GWAS effect estimates are subject to relatively high standard errors. Under these circumstances LDSC and SumHer may produce a negative heritability estimate, in which case a valid estimate of the genetic correlation is not possible. We depict the proportion of invalid estimates for LDSC and SumHer across simulation regime and sample size in Figure S4. Only LDSC and SumHer failed to return valid test statistics for smaller samples: this issue did not affect the nonparametric methods, Hoeffding's test and the GPS tests. It could therefore not explain the downward trend in type 1 error rate for the GPS tests.

We have added an explanation of this point to the caption of Figure 2 and to the Verification and comparison as follows:

> "Both SumHer and LDSC failed to produce valid genetic correlation estimates for a proportion of simulated data sets with small case numbers (Fig S4): where SNP effect estimates are imprecise, SumHer and LDSC can return small, imprecise, and sometimes negative heritability estimates which preclude estimation of the genetic correlation."

There is no clear detail about Hoeffding's test. Why they assess the performance of Hoeffding's test? How are they compared with Li et al.? Does it use a transformation of the standard exponential distribution?

We have added a description of Hoeffding's test to Text S2. We chose Hoeffding's test as a comparator as it, like the GPS test, is a nonparametric method which also assesses a null hypothesis of bivariate independence. We make clear in the revised manuscript that it is quite distinct from the GPS test, being a canonical, general test of bivariate independence rather than one particular to the GWAS context.

1. Can the authors consider more comprehensive simulations? In Table 1, the numbers of causal variants are small. The authors should consider 1,000 or 10,000 causal variants that may be realistic in many disease traits.

We added an additional simulation regime, labelled the 'tiny-effect regime', in which the genetic architecture of the synthetic trait consists of 1,000 causal variants with an odds ratio of 1.02 (cf. the odds ratios of 1.05 and 1.2 used for our small- and large-effect regimes). We found that none of the studied methods displayed appreciable power (see below) to detect shared genetic architecture for all but the largest number of cases, 100,000. The data sets thus did not provide a good basis for comparison of the methods. We decided not to proceed with a 10,000-causal variant regime on the basis that all five methods would presumably perform even more poorly than on the 1,000-causal variant regime.

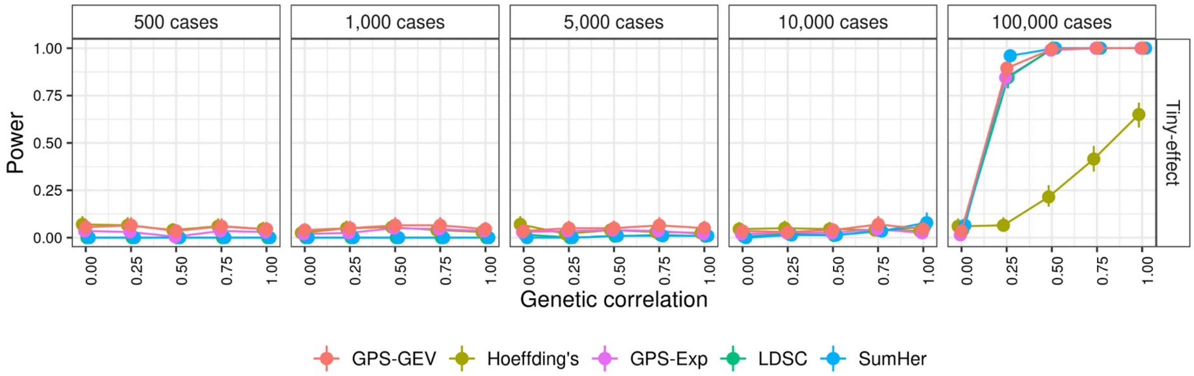

2. The proposed test statistics can used to assess if the genetic correlation is significantly different from 0. Can it be used to test if the genetic correlation is significantly different from 1?

The GPS tests are nonparametric tests, unlike the (Wald) tests of genetic correlation we use for LDSC and Sumer which are parameterised with the value of the genetic correlation under the null, so we cannot specify a null other than one of bivariate independence.

3. Please check references, e.g. there is no journal name in Reference # 33.

We express our thanks for this, we have now corrected several mistakes in the bibliography.

Reviewer 3

In order to test for genetic correlation between two traits with two corresponding GWAS summary data, the authors proposed applying an existing bivariate nonparametric method, called GPS, to test the dependence between two sets of the genome-wide p-values for the two traits, as an alternative to the more common approaches of estimating and then testing the genetic correlation directly as implemented in the popular LDSC and SumHer methods/software. A main contribution is to use a GEVD as the null distribution of the test statistic, instead of an exponential distribution in the original GPS paper. Perhaps the most important and interesting message is that, for small sample sizes (i.e. small numbers of cases) with rare diseases, the proposed GPS performed better than LDSC and SumHer in simulations and an application to the UKB GWAS data. The paper was well written, and if the main conclusion of the paper holds, the proposed method will be useful in practice.

Major comments

1. UKB application: since all the GWAS data/traits are from the UKB, due to overlapping samples, isn't it expected that the p-values of two traits are possibly non-independent? If so, it would undermine the conclusion of the proposed GPS test. From the methods point, the proposed GPS test requires the two GWAS datasets (or p-values) to be independent, doesn't it?

The sharing of cases and controls between UK Biobank GWAS induces a correlation between their test statistics, but this correlation is inversely related to the root of the total sample size of each study[1]. Given the large sample sizes of the studies we used, we expect this correlation and its effect on the GPS tests to be negligible.

2. The null distribution of the GPS statistic: In Fig 1 the authors used the UKB data to show empirically a better approximation of the proposed GEVD over the previously proposed EXP(1); but given the limitations/problems with the use of the UKB data, it may be questionable. Since this is the foundation of the proposed method and a major contribution of the paper, while I suppose that there is no theory to support the use of a GEVD or EXP(1) as the null distribution, some more convincing and extensive evaluations would be helpful.

We repeated this exercise using simulated data sets and found results very similar to those observed with the UKBB data sets. We have added two panels to Figure 1 depicting these new results.

We have added the following sentence to the passage in Verification and comparison treating the choice of null distribution:

> "We observed that the GEVD provided a better fit to p-values from data simulated under the null of bivariate independence also."

Relatedly, given that the SNPs are correlated (with the use of the threshold r2=0.2), strictly speaking, even under H0, a permutational distribution is not necessarily the same as the true null distribution because a permutation would destroy the original correlation structures among the p-value pairs; this point can be addressed in simulations to compare the empirical null distribution of the test statistic (when the data are simulated under H0) with the corresponding permutational distribution. It is also somewhat concerning to see that in Fig 2 the type I error of the GPS test goes down so much as the #cases increases, while those for other methods approach the nominal level as expected.

This is a valid concern and is related to an issue raised by Reviewer 2 regarding the declining trend of the GPS test's type 1 error rate with increasing sample. We refer back to our reply to Reviewer 2 and also to Text S3 ('Type 1 error and sample size for the GPS tests').

In short, we found that our permutation procedure did indeed fail to recapitulate the behaviour of empirical null data, i.e. directly simulated null data, at larger sample sizes, producing sets of null realisations with larger maxima than those seen in the empirical data. This resulted in larger GEV scale parameter estimates and GEV null distributions which yielded overly conservative p-values. This shortcoming of our permutation procedure was mitigated by its only occurring for very large sample sizes uncommon in GWAS. Additionally, we found the GPS test to perform best in the small-sample regime and recommend its use for such data sets. Where the sample size is large enough to result in conservative GPS test p-values, the use of a parametric method like the estimation of the genetic correlation is likely to be preferred in any case for its supplying a meaningful biological parameter, unlike the nonparametric GPS test.

3. How do the test results depend on the LD threshold (r2= 0.2) used in pruning the SNPs? Can the authors do some sensitivity analyses in simulations and real data? This is also related to the reliability of the conclusions with the use of the MHC region in the real data analysis.

We investigated three values of r2 (0.2, 0.5, and 0.8) using single-chromosome simulations under two simulation regimes. We chose to simulate only chromosome 1 rather than the entire genome to reduce the computational cost of this simulation study. We conducted simulations under 'small-effect' (odds ratio 1.05) and 'large-effect' (odds ratio 1.2) regimes with 60 and 20 causal variants, respectively (Table S4).

We found this to be a worthwhile exercise: for r2 = 0.5 and 0.8, both GPS tests (that of Li et al. and our own proposed elaboration, the 'GPS-GEV test') controlled the type 1 error rate conservatively as before but benefitted from an increase in power. Hoeffding's test assumes independent samples (i.e. SNPs in linkage equilibrium) and type 1 error rate was dramatically inflated for r2 = 0.5 and r2 = 0.8. The increase in power for all three tests is visible in the figure below (Fig 4):

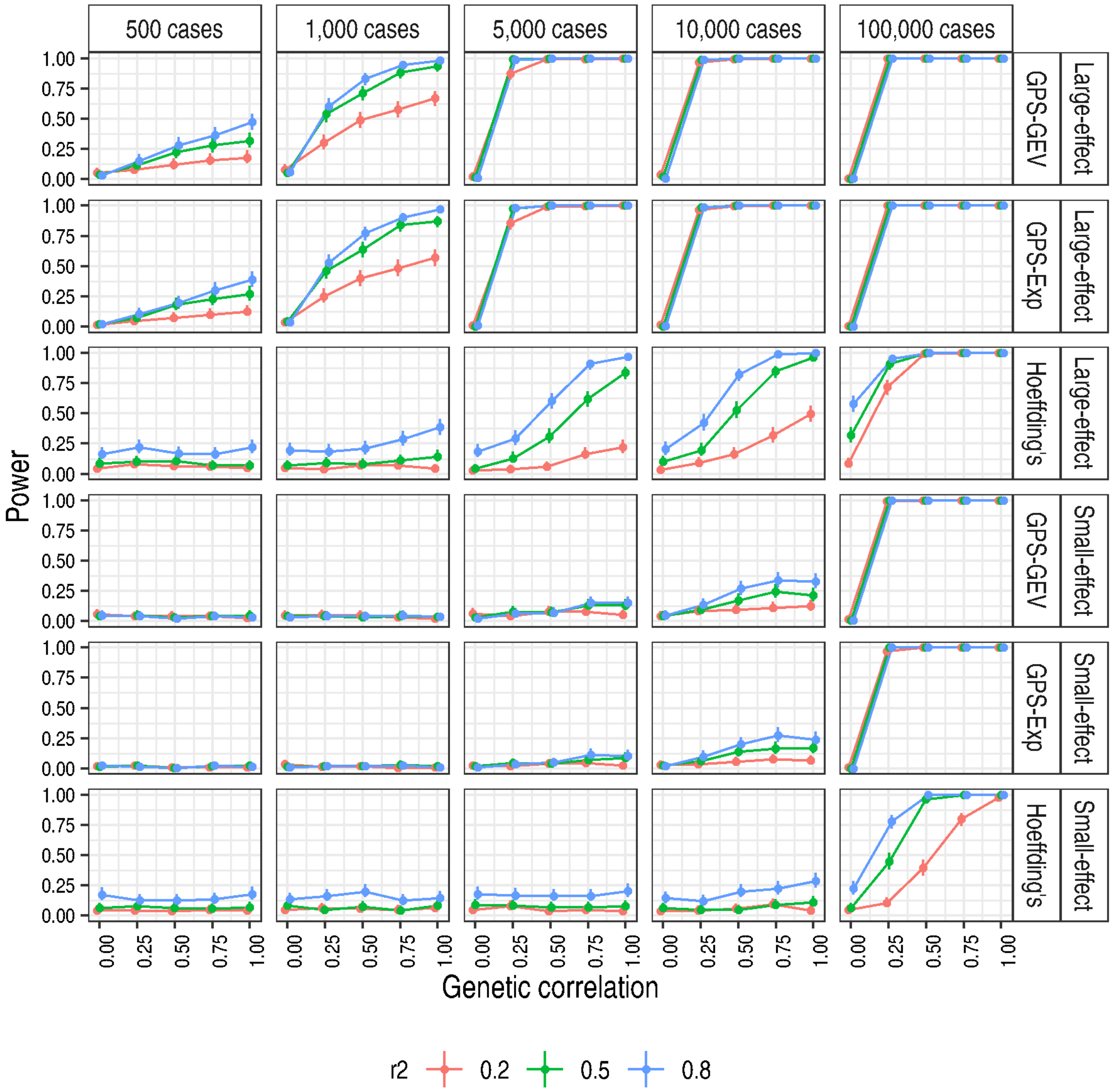

We have expanded our treatment of simulation studies in Verification and comparison with a section describing these single-chromosome r2 studies. As we discuss in the revised manuscript, these results informed our choice of r2 = 0.8 for the analysis of the real data sets from the UK Biobank.

Bibliography

1. Lin, D.-Y. & Sullivan, P. F. Meta-Analysis of Genome-wide Association Studies with Overlapping Subjects. *Am. J. Hum. Genet.* **85**, 862–872 (2009).
