# Supplementary material for: Accurate detection of shared genetic architecture from GWAS summary statistics in the small-sample context
Source: PLoS Genet. 2023 Aug 16;19(8):e1010852. doi: 10.1371/journal.pgen.1010852 (PMC10461826; doi:10.1371/journal.pgen.1010852)
Supplement: Attachment — Submitted filename: response_to_round2.pdf [file pgen.1010852.s016.pdf]

Dear Editors,

Re: PLOS Genetics (PGENETICS-D-22-01171R1)

Thank you for the response to our revised manuscript. We have further revised the manuscript, and provide a point-by-point response below.

Editors' comments:

While reviewers 1 and 2 were generally satisfied with the revision, reviewer 3 remains concerned about the impact of sample overlap on the validity of findings and we feel that this topic warrants further response. Specifically we suggest:

1. Reproduce equation (7) in the Lin and Sullivan AJHG paper that you cite (link here: https://www.ncbi.nlm.nih.gov/pmc/articles/PMC2790578/).
2. Based on this equation and the UKB case-control numbers in Table 2, report the distribution of correlation among effect sizes due to sample overlap across all different combination of traits considered.
3. Perform a simulation exploring the validity of the method under sample overlap at least as great as the "worst" case from 2 above.

The most plausible sample overlap scenario mainly involves controls and we agree this would give little cause for concern. However, shared cases (comorbid phenotypes) can also arise and their effect can be noticeable based on the formula of Lin and Sullivan. This scenario needs to be addressed, briefly but with more detail than currently offered.

> We have performed a simulation following steps 1-3 as suggested. The Lin and Sullvan equation suggests that in our UKBB trait pairs, the expected correlation due to sample overlap (both case and control) ranges from 0.003–0.228, median 0.034 (Fig S9 and Data S4). We performed simulations as proposed and found that only in simulations with very weak effects (less than one genome-wide significant result expected), did increasing correlation increase the type 1 error rate, i.e. when correlation > 0.1 (Fig S10). In data sets with larger effects, the phenomenon we previously noted, of decreasing type 1 error with increasing sample size, was again seen (this time, decreasing type 1 error with increasing power), which overwhelmed any effect of correlation. We understand this to arise in the case of null datasets when none of the strong effects in either dataset line up in the null data, but will do in some of the permutations, producing a more extreme distribution of the supremum test statistic. This means that the test is likely to be conservative in real world use, even in the presence of sample overlap. However, while we appreciate that this point needed further exploration given our application to UK Biobank datasets, we would like to make clear that we chose the UK Biobank datasets only because that was a scenario where we could compare the GPS test to the heritability-based methods. We do not propose that the GPS test should be used where sample sizes are large enough for heritability-based methods to be valid. Instead, we propose that the GPS is useful for those smaller datasets, typically relating to a rare disease, where heritability-based methods cannot be used. In that case, we anticipate that the issue of sample overlap is much less likely to arise, which is why our treatment of this issue was less thorough in our previous response. We have now made this point in the discussion: "We note that, despite our exploration of the effect of sample overlap on performance, we do not anticipate this will be common in its use case, which is likely to involve at least one bespoke GWAS of a rare disease. When samples are large enough to allow heritability-based approaches to be used, we would advocate them,

particularly where they attempt to account explicitly for sample overlap as do LDSC and SumHer." The results summarised above are presented in a new final paragraph in the results (not reproduced here because of the mathematical formatting).

Reviewer's **Comments to the Authors:**

Reviewer #1: The authors have comprehensively answered all my comments, thank you. It seems they have also comprehensively answered the comments of the other two reviewers.

Reviewer #2: The authors have addressed most of my concerns. However, based on the new results, there is no clear evidence that the proposed method outperforms existing methods such as SumHer for moderately and highly polygenic traits, as depicted in Fig. 3 with small effect sizes. Additionally, the traits listed in Table 2 related to the immune system appear to be less polygenic than other complex traits (e.g. type 1 diabetes, RA, etc.). Although I believe this work is suitable for publication, it is important that the authors acknowledge this limitation in their Discussion section to prevent potential confusion among readers and users of the proposed method.

> We agree the method does not outperform SumHer etc in the context of large sample sizes. The intended use-case for the GPS is where SumHer and LDSC cannot be used due to small sample size. We now state this in the last paragraph of the Discussion: "We recommend the GPS-GEV test for the detection of shared genetic effects with small-sample data sets where heritability-based methods are not suitable."

Reviewer #3: I appreciate the authors' responses to my questions/comments. I am fine with most of them except Comment 1, which is about whether/how to account for overlapping samples with the use of the UKB data. Although the authors agree that the overlapping samples would cause non-independence of the sets of the p-values being tested, but stated that
"but this correlation is inversely related to the root of the total sample size of each study 1. Given the large sample sizes of the studies we used, we expect this correlation and its effect on the GPS tests to be negligible."
I disagree. First, because any two traits were measured from (almost) the same set of the UKB individuals, the overlapping proportion is essentially 1. Hence, no matter how large the sample size, this correlation will NOT disappear. Second, as clearly stated in the cited reference 1 (Lin and Sullivan, AJHG, 2009), "failure to account for overlapping subjects can greatly inflate type I error". I do not understand why/how the authors claim that "we expect this correlation and its effect on the GPS tests to be negligible"; I'd love to see both theoretical and empirical/simulation results to support their claim. Note that this is a key issue that may implicate the validity or invalidity of the proposed method (regarding to whether it can control the type I error), but has NOT yet been addressed either theoretically or empirically.

> We appreciate your request for a more detailed response and have followed the editors' request above to provide an empirical analysis. Our expectation that it will have a negligible impact on the UK Biobank studies used here is because we are not performing direct meta-analysis (in which we agree with Lin and Sullivan that ignoring correlation would inflate type

1 error rate), but instead looking at the distribution at the extremum of a combined statistic. We see, reassuringly, that our expectation is largely supported by the empirical results.

However, we also gave this response less detailed attention in our previous response because we think it is less likely to impact the real-world use of GPS. We used here biobank scale data for comparison with other heritability-based approaches. When sample sizes are large enough for heritability approaches to be employed, we would not advocate use of GPS. Its utility lies in analysis of small datasets, often relating to bespoke GWAS of rare traits. In this case, it is unlikely that there would be substantial overlap in controls between the bespoke GWAS and other published studies (biobank or not). We have added some sentences to the discussion emphasising that we do not anticipate GPS being useful in biobank-scale datasets:

“We note that, despite our exploration of the effect of sample overlap on performance, we do not anticipate this will be common in its use case, which is likely to involve at least one bespoke GWAS of a rare disease. When samples are large enough to allow heritability-based approaches to be used, we would advocate them, particularly where they attempt to account explicitly for sample overlap as do LDSC and SumHer.”
